# Endoscopic Mucosotomy and Lumen-Apposing Metal Stent Placement for the Management of a Closed Colorectal Anastomosis

**DOI:** 10.14309/crj.0000000000000616

**Published:** 2021-07-21

**Authors:** Sergio A. Sánchez-Luna, Zain A. Sobani, Tarun Rustagi

**Affiliations:** 1 Division of Gastroenterology and Hepatology, Department of Internal Medicine, University of New Mexico School of Medicine, Albuquerque, NM

## CASE REPORT

**Figure 1.**
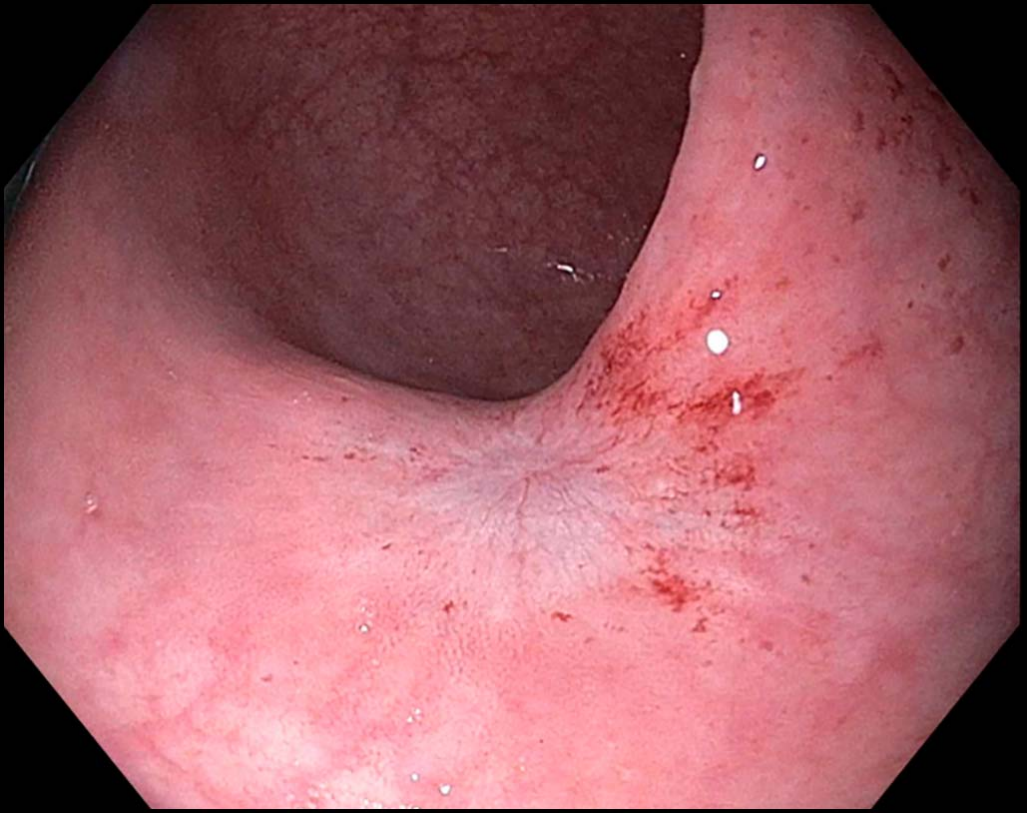
Sigmoidoscopy showed scar at the site of completely closed anastomosis.

**Figure 2.**
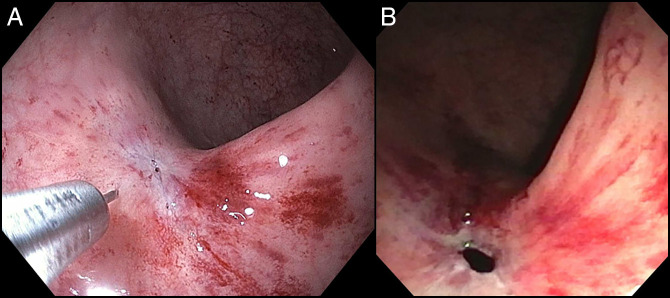
(A and B) Endoscopic mucosotomy was performed with injection needle, followed by endoincision.

**Figure 3.**
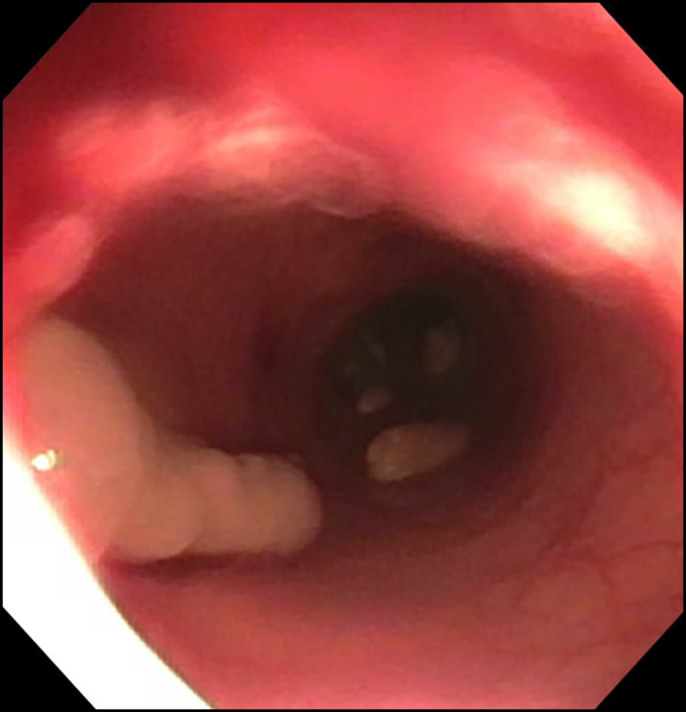
Upstream colon was visualized using an ultrathin endoscope passed through the mucosotomy.

**Figure 4.**
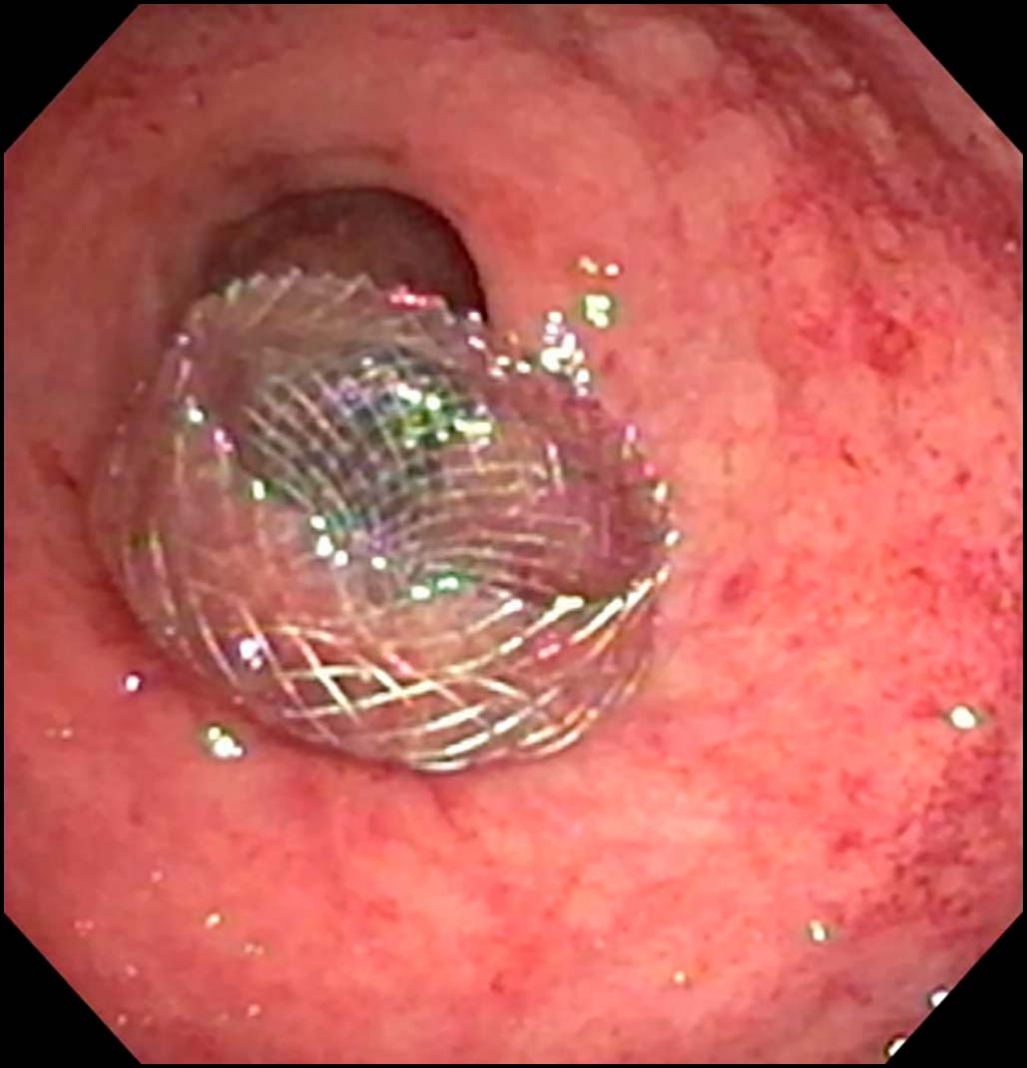
Lumen-apposing metal stent was placed across the anastomotic stricture.

**Figure 5.**
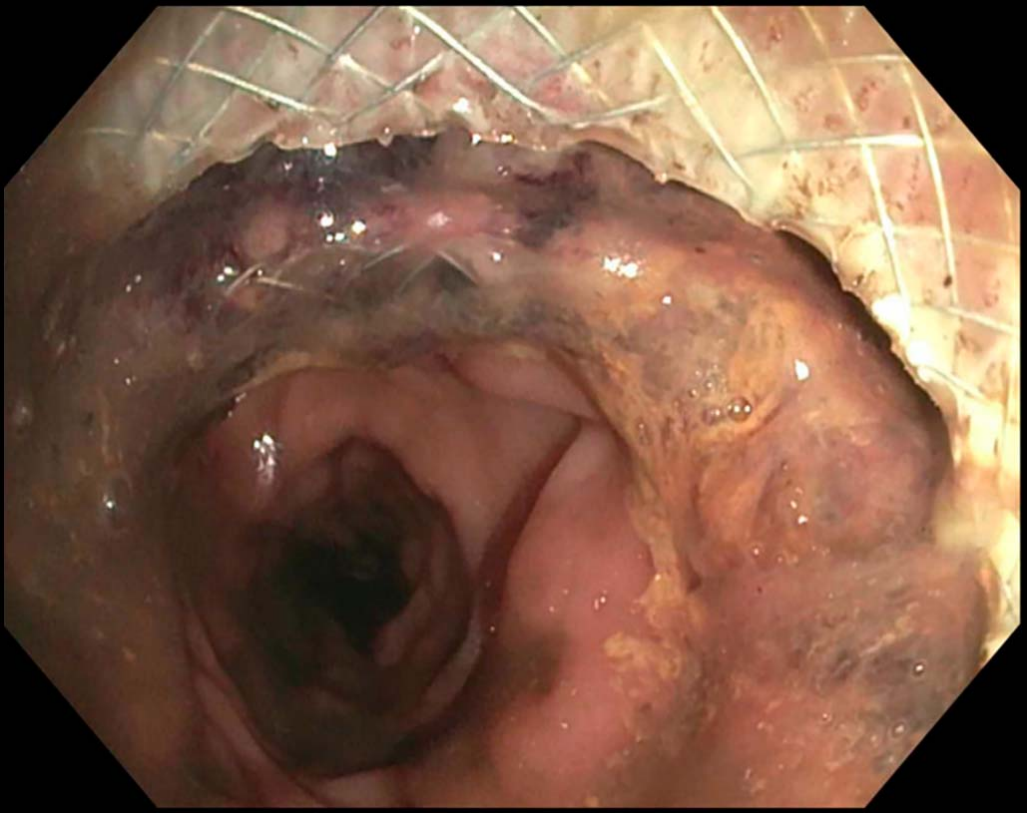
Repeat endoscopy at 7 weeks showed lumen-apposing metal stent in place with a widely patent anastomosis.

A 58-year-old woman with a medical history of metastatic ovarian cancer status post debulking, low anterior resection, and diverting loop ileostomy was referred for a flexible sigmoidoscopy before reversal of her ileostomy 8 months after the surgery. Barium enema showed complete obstruction of the colonic anastomosis. Sigmoidoscopy revealed a scar at the site of anastomosis (5 cm from the anal verge), suggesting a complete obstruction of the anastomosis with overlying epithelialization (Figure 1). Endoscopic mucosotomy was performed with puncturing of the overlying mucosa using an injection needle, followed by endoincision using a needle knife (Figure 2). An ultrathin endoscope was passed through the mucosotomy with visualization and confirmation of the upstream colonic lumen (Figure 3). A 20 × 10 mm lumen-apposing metal stent (LAMS; Boston Scientific, Marlborough, MA) was then placed across the anastomotic stricture to re-establish the luminal continuity and effectively dilate the stricture to 20 mm (Figure 4). Repeat endoscopy 7 weeks after the procedure showed a widely patent anastomosis (Figure 5). The LAMS was removed, and the anastomosis remained patent. The patient had no procedure-related adverse events and underwent reversal of her diverting ileostomy 2 months after removal of the LAMS.

## DISCLOSURES

Author contributions: All authors contributed equally to this manuscript. T. Rustagi is the article guarantor.

Financial disclosure: T. Rustagi is a consultant for Boston Scientific, but this was not relevant to this manuscript.

Informed consent was obtained for this case report.

